# Supplementary material for: Optimal network sizes for most robust Turing patterns
Source: Sci Rep. 2025 Jan 23;15:2948. doi: 10.1038/s41598-025-86854-7 (PMC11757753; doi:10.1038/s41598-025-86854-7)
Supplement: Supplementary file 1 — Supplementary Information. [file 41598_2025_86854_MOESM1_ESM.pdf]

Supplementary Materials:
Optimal network sizes for most robust Turing patterns

Hazlam S. Ahmad Shaberi*[1,2,3], Aibek Kappassov†[1,2], Antonio Matas-Gil[1,2], and Robert G. Endres[1,2]

[1]Department of Life Sciences, Imperial College, London SW7 2AZ, United Kingdom
[2]Center for Integrative Systems Biology and Bioinformatics, Imperial College, London SW7 2AZ, United Kingdom
[3]Institute of Systems Biology, National University of Malaysia

December 4, 2024

Supplementary Figures

In this section, we list additional plots: Figure S1 extends Fig. 3A of the main text by showing empirical histograms of non-Turing Jacobian matrix elements in orange. Figures S2 and 3 extend Fig. 7 on the optimal network size for random Jacobian matrices with sparsity, i.e. missing network links, showing robustness of our key results. Figures S4 and S5 further complement Fig. 7, investigating the role of self-interactions and degradation in the diagonal matrix elements. Figure S6 complements Fig. 8, showing profiles across the equal-diffusivity line for all-Turing (top) and Turing I (bottom). Especially for Turing I, there is a gap in Turing frequency for small N. Finally, Fig. S7 shows the all-Turing case of the Turing heatmap in D_1/D_2 space (instead of just Turing I). Both Figs. S6 and S7 demonstrate that there is no requirement of differential diffusivity for large matrices with many immobile species. Figures S8-S11 show results on the eigenvalue spectra, projected onto the real axis, especially around zero (marginal stability). An optimal network size for Turing patterns emerges based on scaling arguments.

Supplementary Text

Asymptotic circular law and small-N effects

Here, we motivate the emergence of an optimal network size N_{opt}, refining the main text explanation. For small N, the eigenvalue spectrum deviates significantly from the predictions of the asymptotic circular law. These deviations include nonuniform distribution of eigenvalues, with density typically being peaked toward the center of the spectrum. Furthermore, correlations between eigenvalues become more pronounced, especially near the edges of the spectrum, where fluctuations due to finite size lead to deviations from the semicircular profile. These effects are not accounted for in the asymptotic circular law, which assumes infinite matrix size [1] [2] [3].

Asymptotic eigenvalue spectrum. The eigenvalue spectrum for Gaussian random matrices (Wigner Ensemble) describes the eigenvalue distribution of a random $N \times N$ matrix with i.i.d. entries (mean 0, variance σ^2), scaled by $\sigma = 1/\sqrt{N}$. In the asymptotic limit ($N \to \infty$), this gives

$$\rho_{\text{real}}(x) = \frac{1}{\pi}\sqrt{\gamma^2 - (x+1)^2},$$

where $\gamma = \sigma\sqrt{N}$ is the radius of the eigenvalue disk, and the density is non-zero only for $|x+1| < \gamma$. This also approximates Robert May-type random matrices with -1 on the diagonals, which shifts the

*These authors contributed equally to this work.
†These authors contributed equally to this work.

center of the spectrum to -1 on real axis. In our study, the eigenvalue radius is further pinned to $\gamma = 1$ due to Robert May's circular law for all N due to the conditions imposed for a Turing instability.

Finite-N smoothing. For finite N, deviations from the circular law arise due to edge effects and eigenvalue correlations. To account for these, we introduce a smoothing correction:

$$\rho_{\text{smooth}}(x) = \frac{1}{\pi}\sqrt{\max(0, \gamma^2 - (x+1)^2)} \cdot \exp\left(-\frac{(x+1)^2}{2\gamma^2 N}\right), \tag{1}$$

where $\exp(-\frac{(x+1)^2}{2\gamma^2 N})$ accounts for finite-N smoothing. The smoothing factor diminishes as $N \to \infty$, restoring the asymptotic form.

Density at $x = 0$. In the asymptotic case ($N \to \infty$), the density at $x = 0$ is given by

$$\rho(0) = \frac{1}{\pi}\sqrt{\gamma^2 - 1}. \tag{2}$$

For $\gamma = 1$, this evaluates to zero, reflecting the edge of the eigenvalue disk. When finite-N effects are included, the density at $x = 0$ is modified by the above introduced smoothing factor:

$$\rho(0) = \frac{1}{\pi}\sqrt{\max(0, \gamma^2 - 1)} \cdot \exp\left(-\frac{1}{2\gamma^2 N}\right). \tag{3}$$

For small N, the smoothing factor significantly reduces the density, but as $N \to \infty$, it approaches the asymptotic value, Eq. 2.

As a consequence, the impact of N on the spectrum and density are as follows: (1) For small N, the eigenvalue distribution deviates from the perfect circle predicted by the circular law. These deviations smooth out for large N, converging to the circular law's semicircular density profile. (2) The density at $x = 0$ increases with N due to the diminishing impact of the smoothing factor, ultimately saturating at the asymptotic value for $\gamma > 1$.

For small N, the exponential factor can expanded, leading to

$$\rho(0) \sim \frac{1}{\pi}\sqrt{\gamma^2 - 1} \cdot \left(1 - \frac{1}{2\gamma^2 N} + \mathcal{O}\left(\frac{1}{N^2}\right)\right).$$

This scaling shows that as N increases, the density approaches the asymptotic value with corrections diminishing as $1/N$. In particular, the eigenvalue density increases near $x = 0$, i.e. for the marginal stable cases important for Turing pattern formation.

Theoretical prediction for density of Turing cases

To predict the density of Turing cases as a function of N, we assume: (1) the eigenvalue spectrum follows the circular law, and the spectral radius is pinned at $\gamma = 1$. (2) The distribution of σ^2 is Gaussian, with mean $\mu = 1/N$ and variance $\sigma_\sigma^2 \sim 1/N^2$. In random matrix theory, the variance of eigenvalues typically scales inversely with N because the eigenvalues are distributed over a region with a radius scaling as $\sqrt{N}$ (the circular law). The variance of σ^2 (which is proportional to the squared eigenvalue distance) inherits this scaling, leading to $\sigma_\sigma^2 \sim 1/N^2$. For large N, the distribution of eigenvalue fluctuations becomes Gaussian due to the central limit theorem. Hence, the Gaussian distribution for σ^2 is given by:

$$P(\sigma^2; N) \propto \exp\left(-\frac{N^2(\sigma^2 - 1/N)^2}{2}\right).$$

Integrating this over σ^2 gives the density of Turing cases as a function of N only, assuming validity of the circular law (CL) for all N:

$$D_{\text{CL}}(N) \propto \frac{1}{\sqrt{N}}.$$

Finally, including finite-N corrections from the smoothing factor to capture the suppression near $x = 0$, the Turing density becomes

$$D_{\text{Turing}}(N) \propto \frac{1}{\sqrt{N}} \cdot \left(1 - \frac{1}{2\gamma^2 N}\right). \tag{4}$$

This formula captures a possible explanation for the optimal network size N_{opt} observed numerically in the main text. To conclude, the results of this section can be summarized in four plots: Figure S8 shows a comparison of analytical and numerical eigenvalue spectrum as a function of network size N, when projected on real axis. The numerical result shows a peak at the center due to highly unevenly distributed eigenvalues biased towards the center. For increasing N this peak disappears and the density approaches the analytical results are based on Eq. 1 with finite N smoothing. The analytical result approaches the asymptotic limit for large N. Figure S9 shows the analytical eigenvalue spectrum with the details of the finite-N smoothing corrections based on Eq. 1. We see that the density increases right below $x = 0$ on real axis. Furthermore, Fig. S10 focuses on this marginal eigenvalue density near $x = 0$. This shows that density increases with N to approach the asymptotic limit. Finally, in Fig. S11 the Turing density is plotted as a function of N. This shows a maximum for small N due to small-N effects, i.e. suppression of density for marginal stable networks. The analytical results are based on a version of Eq. 4, only valid for small N.

References

[1] Forrester PJ. The spectrum edge of random matrix ensembles. Nuclear Physics B. 1993 Aug;402(3):709-28. Publisher: North-Holland. Available from: https://www.sciencedirect.com/science/article/abs/pii/055032139390126A.

[2] Tao T, Vu V. Random matrices: Universality of local eigenvalue statistics. Acta Mathematica. 2011 Jan;206(1):127-204. Publisher: Institut Mittag-Leffler. Available from: https://projecteuclid.org/journals/acta-mathematica/volume-206/issue-1/Random-matrices-Universality-of-local-eigenvalue-statistics/10.1007/s11511-011-0061-3.full.

[3] Rider B. Deviations from the Circular Law. Probability Theory and Related Fields. 2004 Nov;130(3):337-67. Available from: https://doi.org/10.1007/s00440-004-0355-x.

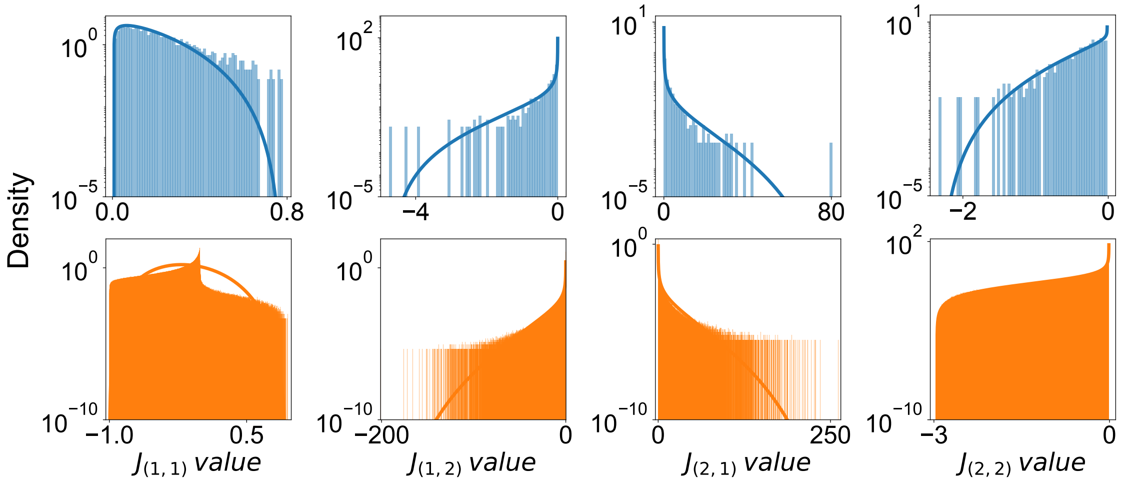

Figure S1: Empirical distributions of Jacobian matrix elements for 2 node network. This extends Fig. 3A, showing histograms for both Turing I instabilities (light blue) and non-Turing (light orange), along with fits to beta distributions for Turing I (blue line) and non-Turing (orange line). Empirical distributions are computed based on parameter sampling for the pre-defined 2-node network topology in Fig. 1, Ai.

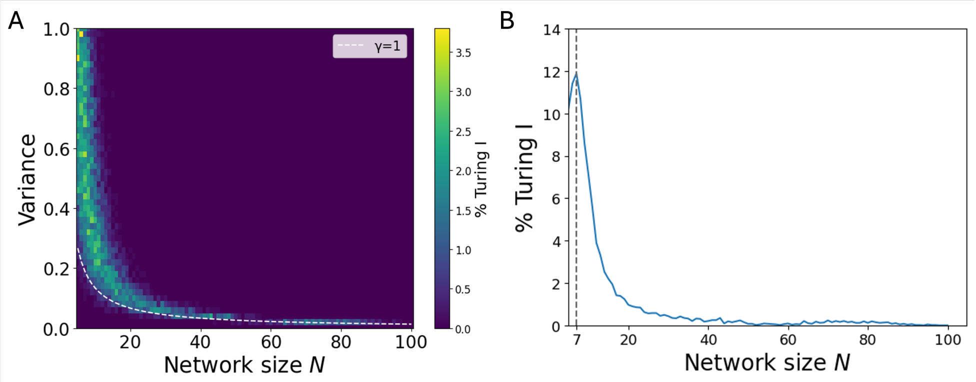

Figure S2: Optimal network size for highest Turing robustness for small sparsity. (A) Heat map of percentage occurrence of Turing I in random matrices of different networks size N and variance σ^2 for $\gamma = 1$ and 25% off-diagonal element sparsity ($C = 0.75$). A dashed white line corresponds to the equation $\sigma^2 = 4/(3N)$. (B) Corresponding percentage shares of each network size N. For our parameters, the optimal network size is $N = 7$. The horizontal axis is set to linear scale.

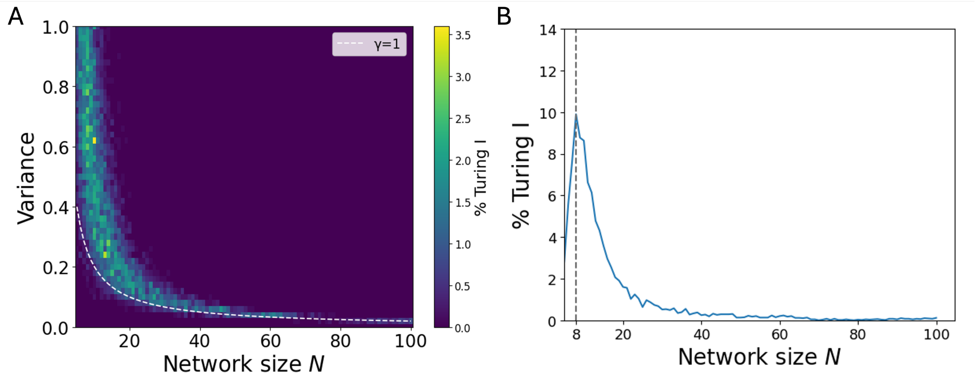

Figure S3: Optimal network size for highest Turing robustness for strong sparsity. (A) Heat map of percentage occurrence of Turing I in random matrices of different networks size N and variance σ^2 for $\gamma = 1$ and 50% off-diagonal element sparsity ($C = 0.50$). A dashed white line corresponds to the equation $\sigma^2 = 2/N$. (B) Corresponding percentage shares of each network size N. For our parameters, the optimal network size is $N = 8$. The horizontal axis is set to linear scale.

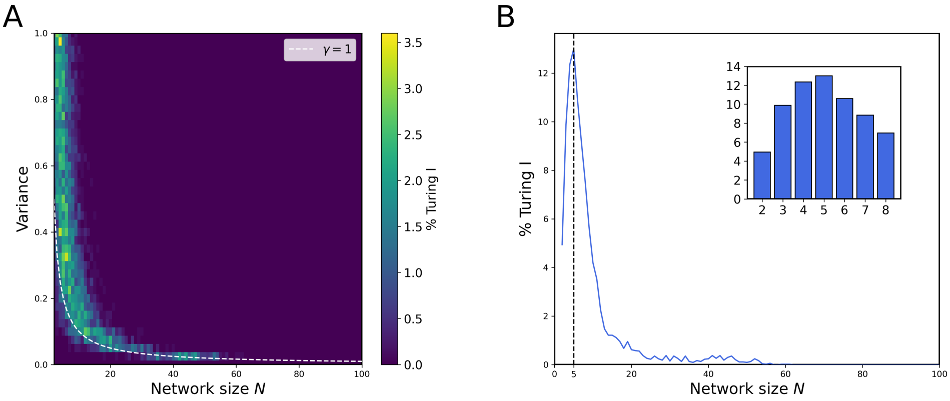

Figure S4: Optimal network size for Turing robustness with self-interactions and fixed species degradation. (A) Heat map of percentage occurrence of Turing I in random matrices of different network sizes N, variance σ^2 (including for diagonal matrix elements), and degradation -1 for $\gamma = 1$ and no sparsity (connectivity $C = 1$). (B) Corresponding percentage shares of each network size N. For our parameters, the optimal network size is $N_{\text{opt}} = 5$. The inset provides a more detailed view of network sizes from 2 to 8 for finer resolution, showing that $N = 2$ is now allowed for pattern formation.

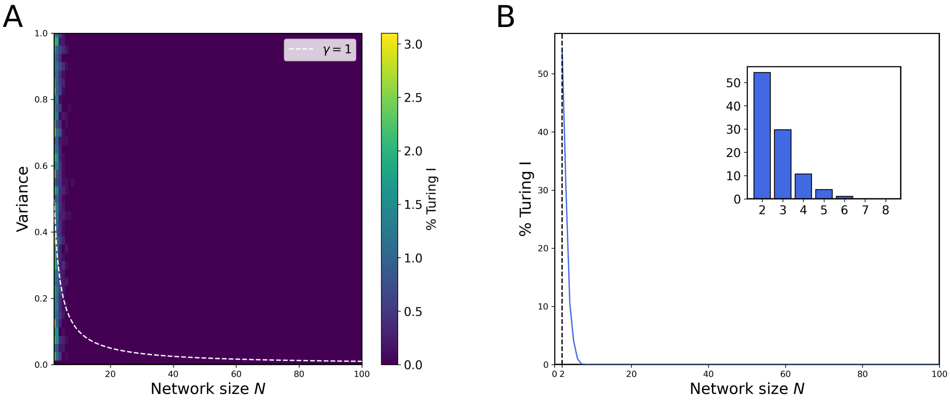

Figure S5: Optimal network size for Turing robustness with self-interactions but no fixed species degradation. (A) Heat map of percentage occurrence of Turing I in random matrices of different network sizes N and variance σ^2 (including for diagonal matrix elements) for $\gamma = 1$. There are no additional degradation and no sparsity (connectivity $C = 1$). (B) Corresponding percentage shares of each network size N. For our parameters, the optimal network size is now reduced to $N_{opt} = 2$, i.e. the smallest allowed network size. The eigenvalue circles are now centered around $x = 0$ on the real axis, with eigenvalue movement by diffusion increasingly eliminated for increasing N, leading to either stable or unstable matrices irrespective of diffusion. The inset provides a more detailed view of network sizes from 2 to 8 for finer resolution.

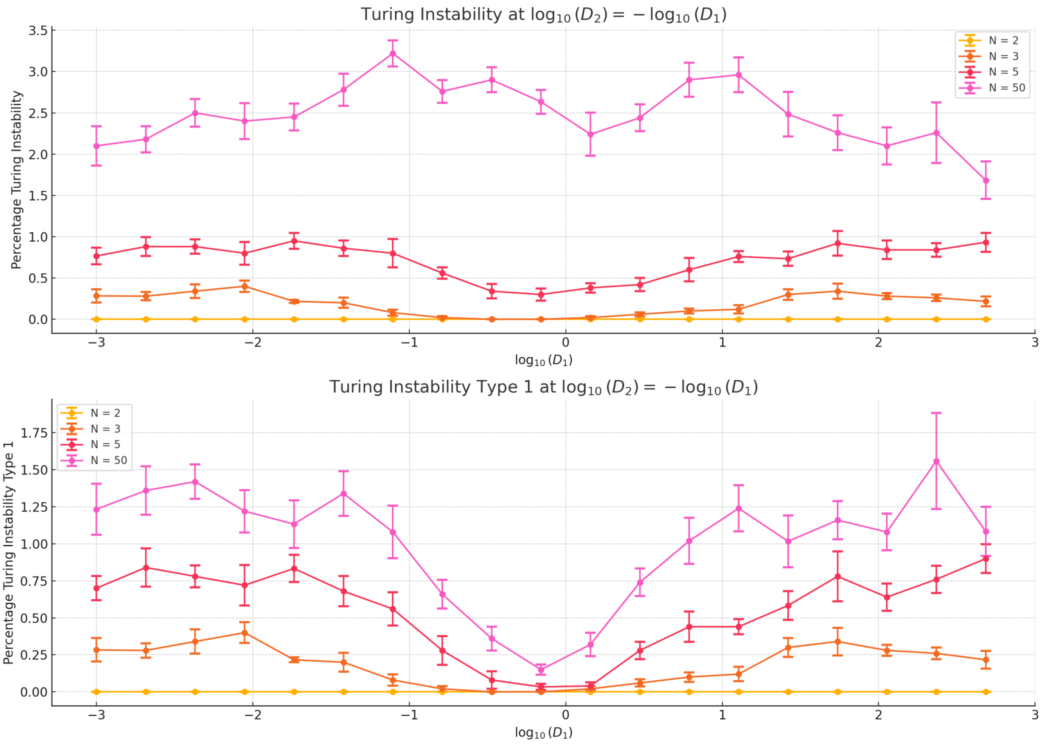

Figure S6: Visualizing the diffusivity gap. Profile plot for data from Figs. 8 and S5. The the heatmap simulation data was filtered and binned around the diagonal line $\log 10(D_2) = -\log 10(D_1)$, showing averages and standard errors of the all-Turing (top) and Turing I (bottom) percentages.

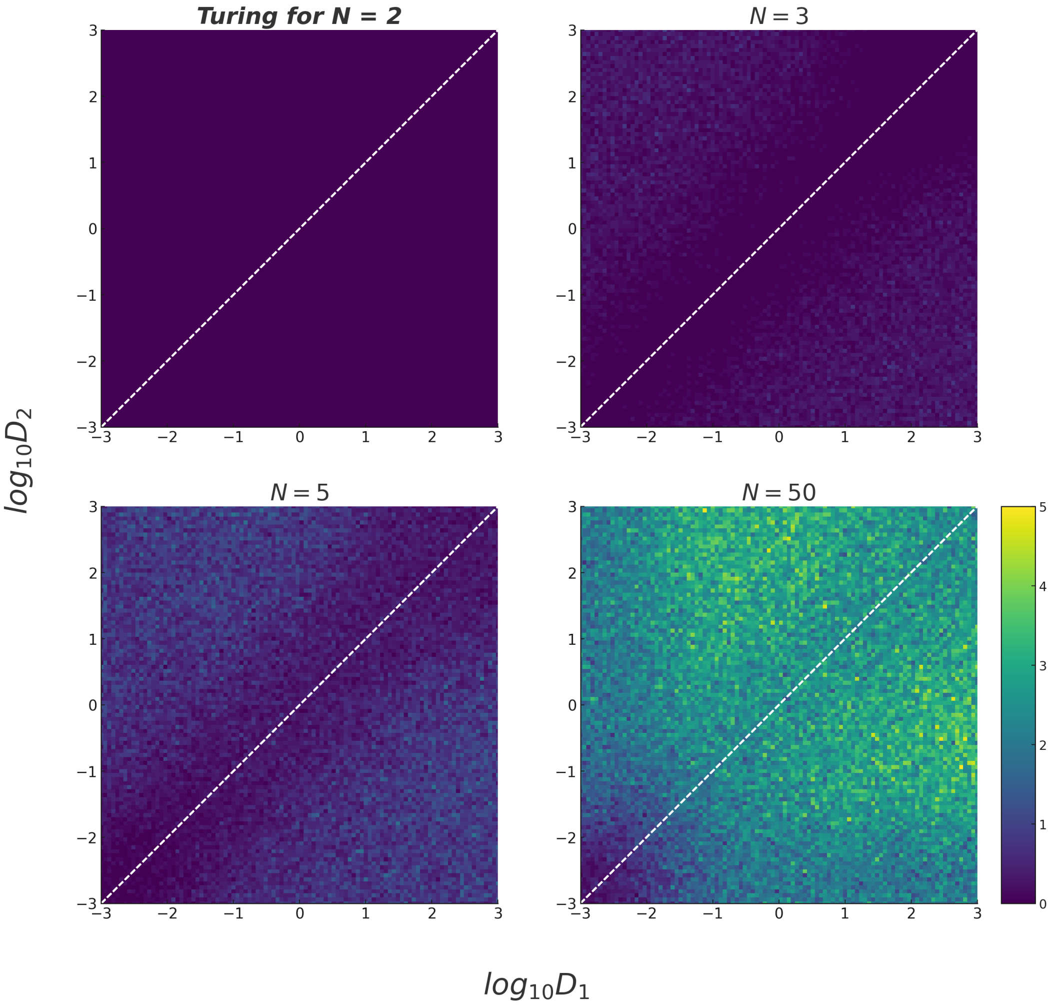

Figure S7: Effect of diffusion constants on Turing pattern formation. Heat map of percentage occurrence of all Turing (I, II, and Turing-Hopf) in random matrices for different diffusion parameters, D_1 and D_2: (A) $N = 2$, $\sigma^2 = 0.5$; (B) $N = 3$, $\sigma^2 = 0.33$; (C) $N = 5$, $\sigma^2 = 0.2$ (D) $N = 50$, $\sigma^2 = 0.02$. For increasing N, the constraint on differential diffusivity vanishes.

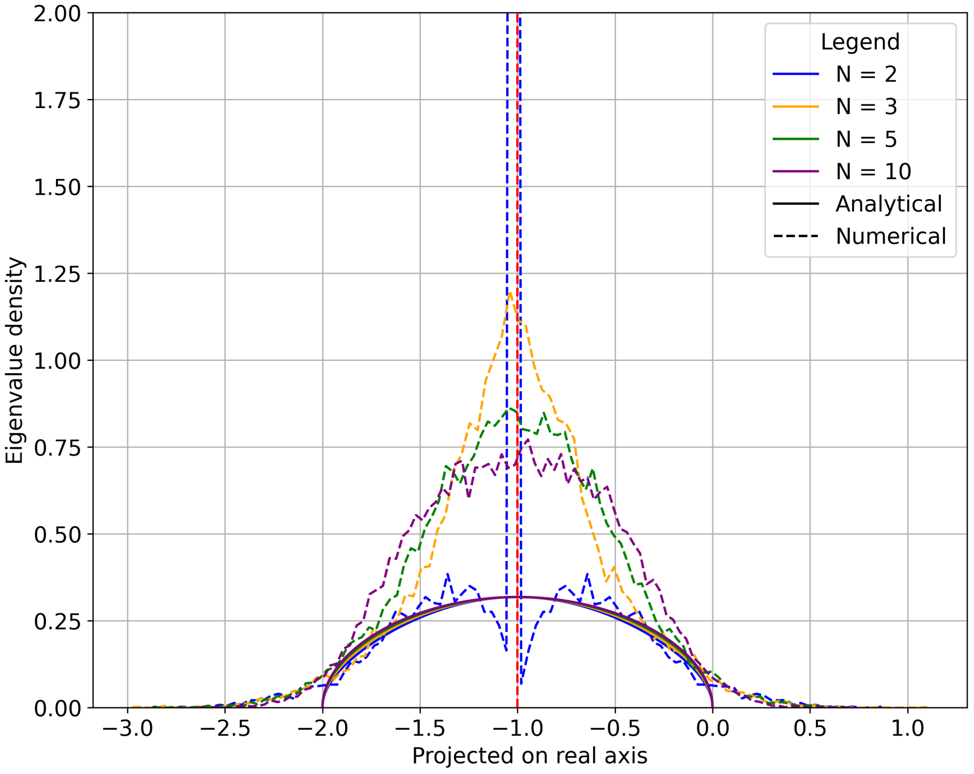

Figure S8: Comparison of analytical and numerical eigenvalue spectra. Spectra as a function of network size N, when projected onto real axis. The analytical results are based on Eq. 1.

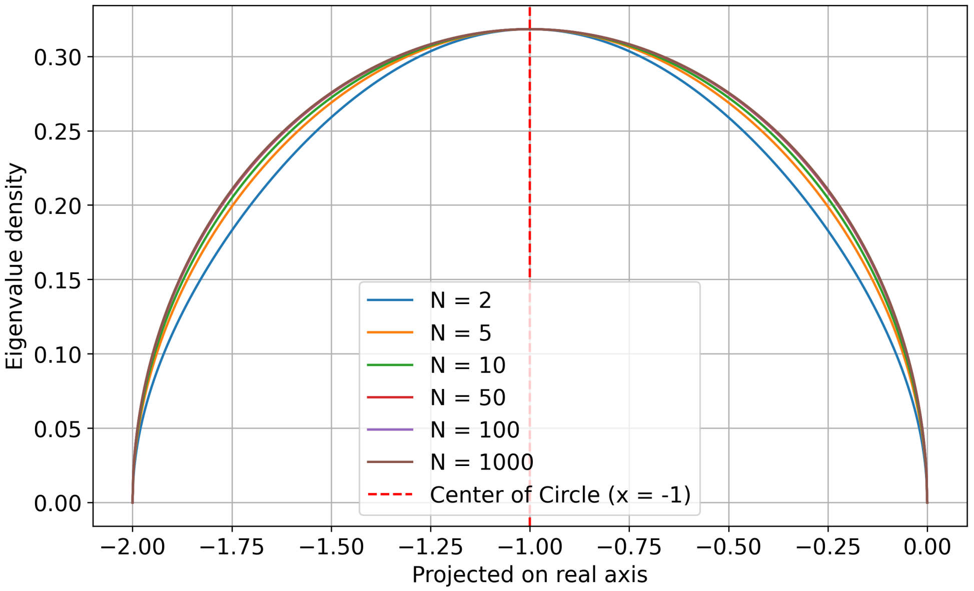

Figure S9: Analytical eigenvalue spectrum with finite-N smoothing corrections. The analytical results are based on Eq. 1.

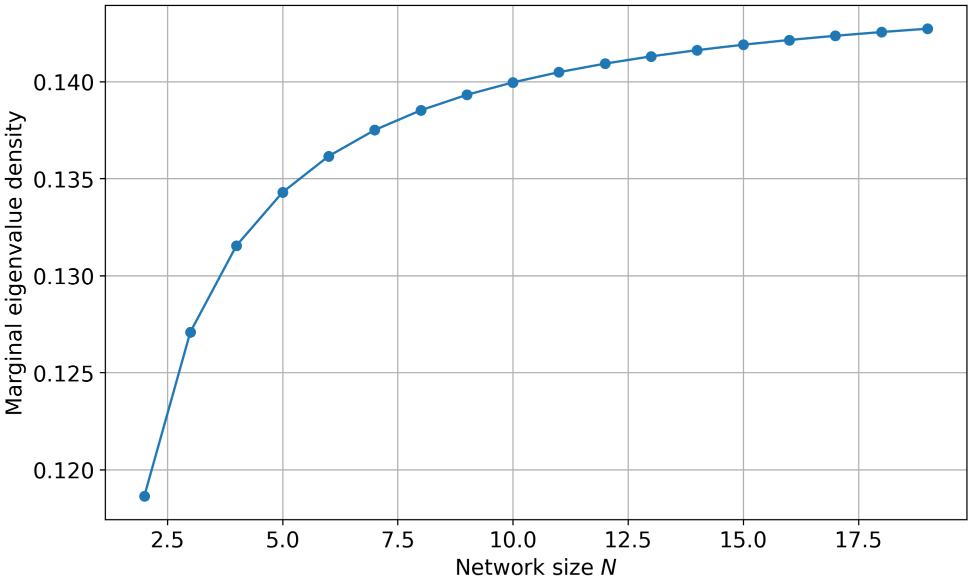

Figure S10: Marginal eigenvalue density around zero on real axis. Density increases with N to approach the asymptotic limit. The analytical results are based on Eq. 2.

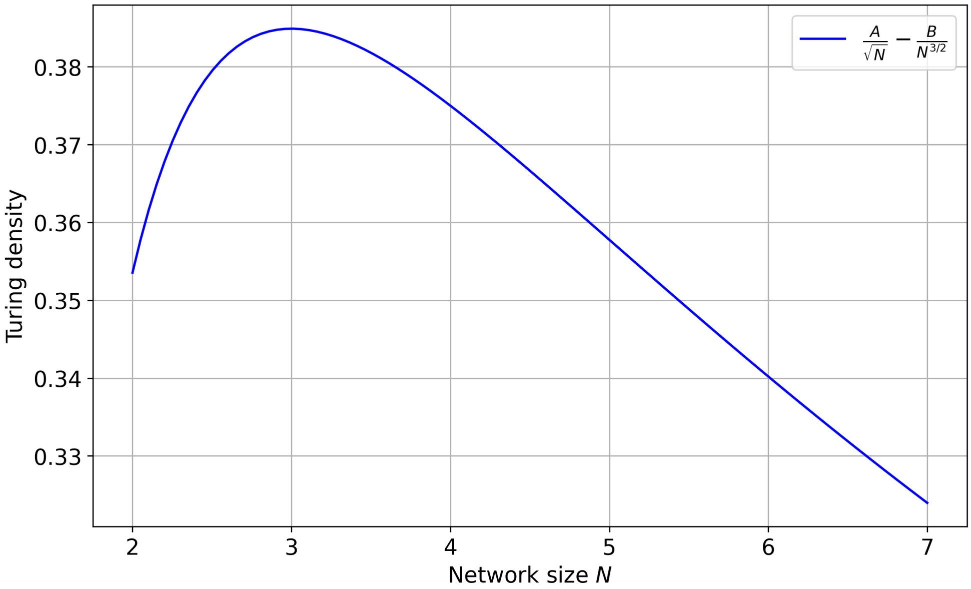

Figure S11: Turing density as a function of N. This shows a maximum for small N due to small-N effects (the exact position of the peak depends on the details of the smoothing function used). The analytical results are based on a generalized Eq. 3, using $A = B = 1$.
